# Student perceptions of COVID-19 challenges affecting student motivation, well-being, and success in undergraduate education

**DOI:** 10.1371/journal.pone.0324832

**Published:** 2025-06-02

**Authors:** Jonathan F. Prather, Dan McCoy, April Heaney, Laurie A. Smith, Eirin Grimes, Natalie A. Bloomston, Nycole Courtney

**Affiliations:** 1 Department of Zoology and Physiology, Life Sciences Program, University of Wyoming, Laramie, Wyoming, United States of America; 2 Haub School of Environment and Natural Resources Program, University of Wyoming, Laramie, Wyoming, United States of America; 3 Learning Resource Network Program, University of Wyoming, Laramie, Wyoming, United States of America; 4 Office of Student Success and Graduation, University of Wyoming, Laramie, Wyoming, United States of America; 5 Counselor Education and Supervision Program, University of Northern Colorado, Greeley, Colorado, United States of America; University of Pretoria, SOUTH AFRICA

## Abstract

Our objectives in this study were to understand the impact of COVID-19 disruptions on the academic and personal experiences of undergraduate students at a state land-grant institution in the Western United States, and to use those insights to identify actionable ways to improve student success. We used a mixed method survey to assess strategies used by undergraduates to adapt to COVID-19 disruptions. Results revealed that despite challenges, the majority of students continued toward their academic goals. Face-to-face classes yielded the greatest student satisfaction, and students reported great dissatisfaction with separation from peers and instructors. These insights will be especially helpful to educators and administrators in responding to future challenges and planning future approaches. This overview of students’ attitudes associated with moving from in-person to online coursework may also be useful for advising students considering which of these instructional paradigms to pursue.

## Introduction

The COVID-19 pandemic had an unprecedented impact on the personal and academic experiences of students worldwide [1]. Students at institutions of higher education across America were leaving higher education or not entering at all, taking fewer classes, juggling academic and caregiving responsibilities, and concerned about their financial, professional, and personal well-being [2]. Reports emerging about how students were affected highlight the negative impacts on factors such as mental health, physical health, and sense of safety, sense of belonging, and insecurity of essential resources such as food, housing, and finances [3–18]. Those reports can help us understand the psychological, emotional, financial and academic toll that COVID-19 took on students. That perspective formed the intellectual framework for this study of how students were affected during the pandemic, what facets of their academic and social environments were most important to their feeling of well-being, and how they adapted their academic approaches to persevere in the face of those challenges [16,17,19].

Broadly defined, student success comprises a suite of factors that enable students to thrive in their educational experience and proceed toward timely degree completion [20,21]. Helping students achieve those goals enables them to persist in their degree programs, whereas difficulties in those areas can lead students to pause their progress, cease their progress altogether, or fail to enroll at the start of that process. Enrollment was down by 560,000 students (a decline of 3.6%) in the Fall 2020 semester immediately after the onset of the pandemic compared to the Fall 2019 semester, and this was especially evident at institutions that serve students with the fewest resources [2]. Current trends toward reduced undergraduate enrollment highlight the necessity for institutions to take a closer look at their methods for enhancing students’ success [22,23]. With the goal of developing targeted ways to help our students achieve their academic goals, we sought to understand the impact of the COVID pandemic and the associated changes in personal and academic contexts on the well-being and continued degree progress of our students.

Developing effective solutions to help students succeed and persist in their degree programs requires institutions to understand students’ experiences. An institutional culture that values dialog between students, faculty, staff, and administrators is essential to identify current strengths, weaknesses, opportunities, and possible ways that those insights can be translated into strategies to enhance student success. In response to the pandemic, leaders at the University of Wyoming reached out to all these groups, with the goal of identifying ways in which some individuals may have thrived because of specific strategies, or ways in which less effective strategies may have contributed to challenges for others. Central to that effort was a survey distributed to all undergraduate students at the University of Wyoming in the Fall semester of 2020. Students’ responses offered a more comprehensive picture of how the pandemic had affected their undergraduate experiences.

We surveyed undergraduate students at a state land-grant university in the Western United States in the months following the onset of the pandemic. We analyzed their responses to identify the impact of those disruptions on students’ perceptions of well-being and success. We also asked them about what aspects of institutional practices were most effective in helping them feel supported and effective in their academic pursuits. Finally, we also asked them about their intention to continue in their studies, and we compared those self-reports to their actual decision of whether to persist in their academic careers. We analyzed students’ responses to understand how the pandemic affected our students’ undergraduate experience and how they developed strategies to cope in response to those unusual challenges. Students’ responses offered a detailed picture of how pandemic-induced changes in instructional approaches affected the students’ undergraduate experiences and how those lessons can be harnessed to improve ongoing educational approaches such as welcoming new students and building online degree programs [17,24].

The insights gained from our analyses point to possible pilot interventions that may be especially effective in helping students forge new and lasting social and professional connections. For example, students highlighted the value of in-person interactions with their peers and instructors. To facilitate those interactions, leaders at our institution have implemented a week-long orientation experience in which students were guided in forming personal connections with peers and instructors. We provide a brief description of that program designed to reach out to help populations that were most impacted. Pandemic-induced adaptations of educational approaches involved a move into the online environment, and this trend persists in the prevalence of online degree programs. Therefore, these lessons will be especially helpful as educators continue to improve the ways in which we engage students through distance learning opportunities [13,14,17]. In the following sections, we describe the survey and the methods that were used to assess the impact of the pandemic on student experiences. We then describe our findings regarding the impact of the pandemic on factors such as student motivation, retention in the following semester, students’ opinions about instructional formats, and the impact of a range of factors on students’ satisfaction and success or struggle during that dynamic time. We conclude with a general consideration of implications of these findings for future efforts to improve academic and social environments as key elements in supporting students’ success and well-being throughout their undergraduate education.

## Methods

### Survey design and data collection

The survey was designed collaboratively by members of Academic Affairs (Administrators and Faculty) and Student Affairs (Administrators and Staff) at the University of Wyoming in September and October of 2020. All questions were composed collaboratively by three of the authors (Jonathan F Prather, Laurie A Smith, and Nycole Courtney). All questions were reviewed and revised by a panel of faculty, staff, students, and administrators, and all members of that panel approved the questions prior to survey distribution. That panel included all of the authors and members of their work teams (12 people total: 5 faculty, 2 staff, 3 students, 2 administrators). The questions were thus vetted by a panel of experts, but the questionnaire was not subjected to pilot testing or external validation prior to distribution.

The survey contained a total of 17 questions and requested students’ written consent to collect and analyze their responses. Students were also asked for identifying information such as name, email address, and demographic information. An additional three questions asked students about whether they intended to persist in their undergraduate education in the following semester at either this institution or elsewhere. Three multi-part questions (five to 12 items in each question) used a Likert scale to ask students about a wide range of aspects of their academic and social experience, and four short-answer questions provided students an unconstrained opportunity to share about facets of their experience such as approaches and adaptation they found helpful to facilitate their success. Together, the questions in the survey addressed several topics including the students’ plans regarding their possible return in the spring semester, the factors influencing those plans, their long-term intent regarding graduation, aspects of academic and personal experiences in which they succeeded or struggled, satisfaction with instructional formats, personal explanations of how they navigated the semester, and the ways in which specific instructional approaches were or were not helpful. The complete text of the full survey is available online through the same link where anonymized data have also been made publicly available (openicpsr-211905; https://doi.org/10.3886/E211905V1). As detailed below, results from the portions of the survey that included categorical or Likert responses (questions 2, 3, and 5) were analyzed quantitatively. The remaining portions of the survey included either consent or demographic information (questions 1 and 6) or qualitative responses (question 4). The Results below were taken from only quantitative data (questions 2, 3 and 5). Qualitative data (question 4) were part of a separate investigation and were not included in this analysis.

The survey and follow-up reminders were distributed via Qualtrics survey software near the end of the Fall 2020 semester (November 5, 2020). The contact email that accompanied the survey indicated that responses were requested within the coming days, and a link was provided to facilitate students accessing and completing the survey. Between the time of initial contact to the end of data collection (November 29, 2020), students had 24 days to complete the survey.

### Ethics statement

The survey was reviewed by the University of Wyoming Institutional Review Board in November 2020 and was determined to be a permitted component of program evaluation. All participants were adults at least 18 years of age, all participants gave written consent for their participation and inclusion in this analysis, and all data were analyzed with the participants’ identity made anonymous.

### Participants and demographics

The survey was distributed to all undergraduate students enrolled at the University of Wyoming during the Fall 2020 semester (9,342 students). Responses were received from 2,307 students (24.7%), with 2,199 (23.5% of students queried, 95.3% of respondents) completing all portions of the survey. Data were collected from students across all undergraduate academic standings (e.g., freshmen, sophomores). Students averaged approximately nine minutes to complete the survey.

The University of Wyoming is a public land-grant University located in Southeast Wyoming in a town that is home to 32,382 residents. Academic opportunities span 192 majors, including 78 Bachelors degree programs, 57 Masters degree programs, 21 certificate programs, and 36 doctorate or professional degree programs. Courses in those programs are offered through both on-site and online courses. The 9,342 undergraduate students queried in this study were part of a community that includes 12,397 undergraduate and graduate students (Table 1). A slight majority of students are female (50.6%), and a majority of students are residents of the state of Wyoming (64.8%). Nonresident students come from all 50 states and 91 countries around the world.

**Table 1:** Demographics of undergraduate students at the University of Wyoming in the Fall 2020 semester.

		Number	Percent
**Enrollment**	Full Time	7783	83.3%
	Part Time	1559	16.7%
**Class Standing**	Freshman	1784	20.1%
	Sophomore	1852	20.9%
	Junior	2153	24.3%
	Senior	3075	34.7%
	Non-degree Undergrads	202	42.3%
	Second Bachelors	276	57.7%
**Residence**	In State	6051	64.8%
	Out of State	3291	35.2%
	Domestic	9146	97.9%
	International	196	2.1%
**Ethnicity**	White	7121	92.5%
	Hispanic, Latino, or Spanish	576	7.5%
	Two or more races	379	4.9%
	Black	107	1.4%
	Asian	106	1.4%
	American Indian or Alaska Native	67	0.9%
	Native Hawaiian/Pacific Islander	10	0.1%
	Not Reported	780	10.1%
**Family History**	First Generation	2899	31.0%
	Continuing Generation	6443	69.0%

These values describe the demographics of the campus community.

Only first generation status was directly queried in the survey.

### Analytic strategy

Student responses were consolidated by one author (L. A. Smith) and all personally identifying information was removed before sharing any information with other authors who performed the analyses. Quantitative analyses included tallying the responses of the entire set of respondents to gain insight into the community’s thoughts regarding the topics in each question. Those responses were also parsed according to academic standing (e.g., freshmen, sophomores) to identify whether some facets of the pandemic experience were more or less impactful across those groups. Results are reported as means [25]. Commentary in the results and discussion is based on comparison of consistent trends in the data (e.g., a difference between groups that is evident across all measures) or broad differences in the influence of specific factors (e.g., some that resulted in positive responses versus others that resulted in negative responses). Statistical comparisons were not performed, as our goal was to detect such broad or categorical distinctions as a way of identifying the most impactful influences on students’ responses.

## Results

### Most students remained motivated to complete their undergraduate education

**Fig 1:**
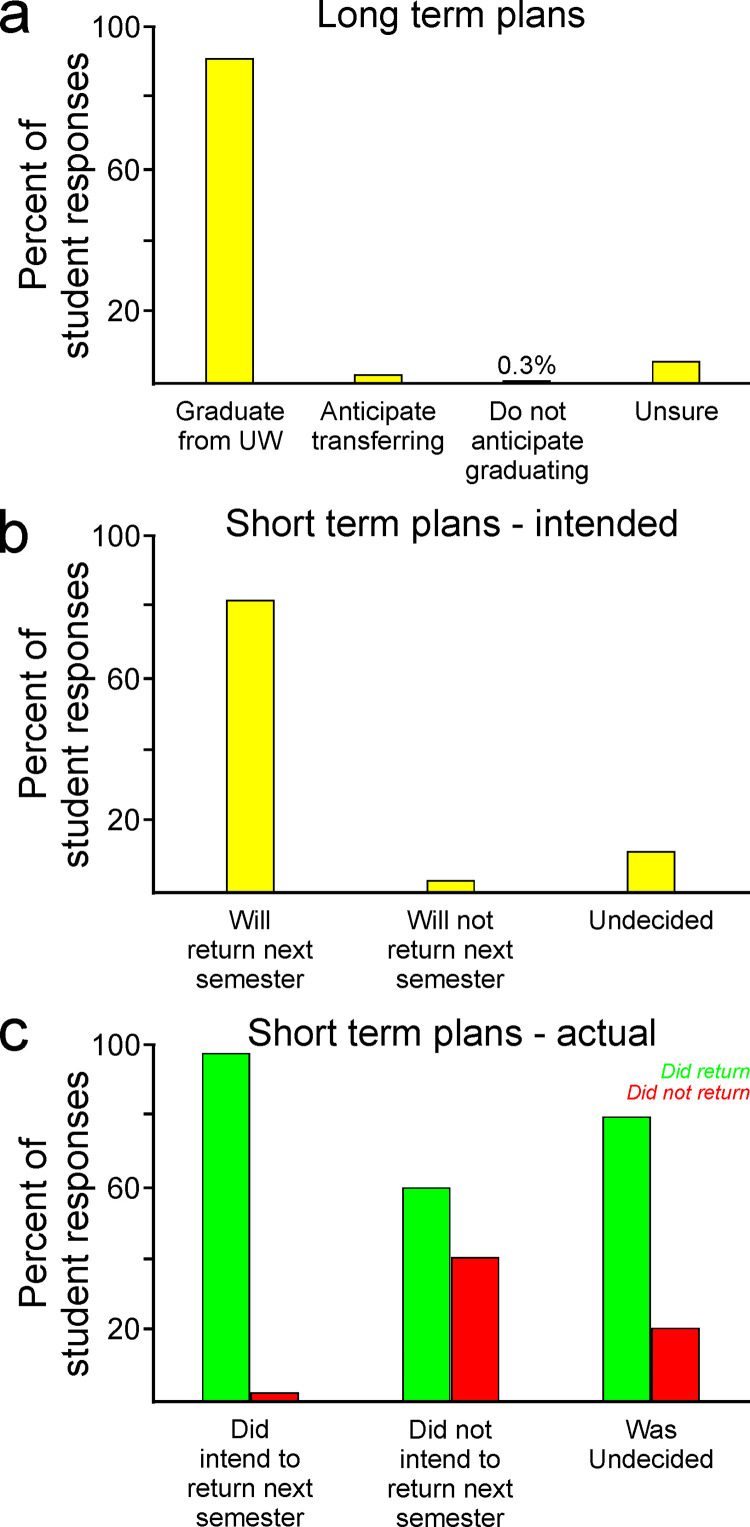
Student’s intentions and actions regarding retention after the onset of the pandemic. Students persevered in their degree progress, as evident in both their (a) long-term and (b) short-term plans. This was also evident in their (c) continuation toward degree completion in the semester following the onset of COVID disruption.

Students remained motivated to graduate even in the face of challenges that emerged in the COVID-19 pandemic (Fig 1A). Ninety-one percent of respondents indicated that they not only intended to graduate but also intended to graduate from their current institution (Fig 1A). Only 2.5% of students indicated that they intended to transfer to another school, and only 0.3% indicated that they did not intend to graduate at all. Together these data highlight that many students retained their motivation to succeed even in the face of unprecedented changes in their educational experience.

### Student retention in the following semester was very high

Students persevered in not only their long-term plans to graduate but also their intention to continue immediately by returning in the following semester (Fig 1B). Among the 2,306 respondents to this question, 82.1% reported that they intended to return in the following semester. In contrast, only 2.9% indicated that they did not intend to return in the following semester, and 10.8% indicated that at the time of the survey they were unsure of their immediate plans.

We also collected enrollment data at the start of the following semester (Spring 2021) to measure the degree to which students followed those intended plans. Among students who indicated an intention to return, retention was very high, with 97.6% of those students returning in the following semester (Fig 1C, left columns). Somewhat surprisingly, even among students who initially indicated that they did not intend to return in the following semester, 59.7% of those students returned and continued uninterrupted in their academic progress (Fig 1C, middle columns). In a result that reflects positively on the students’ overall experience in the Fall 2020 semester, 79.5% of students who indicated they were undecided about whether they would return eventually returned in the Spring 2021 semester (Fig 1C, right columns).

Overall, the rate of retention from the Fall 2020 to the Spring 2021 semesters was 89.7%. In recent years (2015–2019) the average rate of retention at our institution during the transition between Fall and Spring semesters has been 91.7%. The fact that retention was so similar to those historical values, even in the face of such unusual circumstances, suggests that students were resolute in their desire to pursue their degrees and were reasonably satisfied with their experiences in the Fall 2020 semester. In the following sections we describe the results of questions intended to yield greater insights into the role of specific factors in shaping students’ satisfaction and perception of the impact of the pandemic on their educational experience.

### Students adapted to online paradigms but preferred approaches that retained face-to-face elements

**Fig 2:**
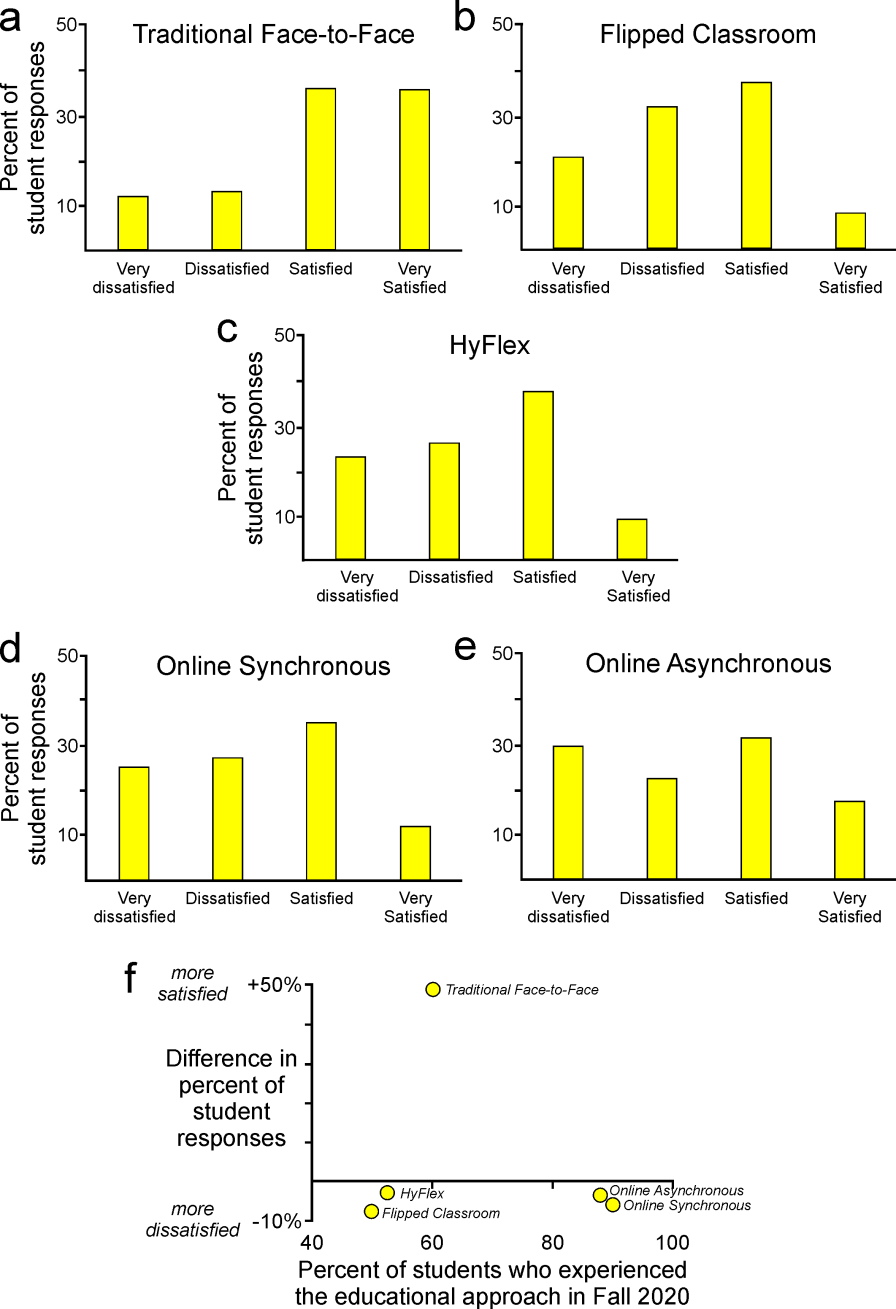
Students’ satisfaction with various instructional formats. (a-e) Students reported varying degrees of satisfaction with different instructional formats, (f) with greatest preference for the familiar face-to-face environment.

Students reported the greatest degree of satisfaction with courses in the traditional face-to-face approach (Fig 2A). That preference was evident as 48.4% more students reported that they were either satisfied or very satisfied (Fig 2A, right two columns) versus the percentage of students that reported that they were either dissatisfied or very dissatisfied (Fig 2A, left two columns). This result was perhaps predictable given the extent to which students were familiar with face-to-face approaches and were perhaps therefore more comfortable in that setting.

Students reported lesser degrees of satisfaction with other approaches. Approaches that incorporated online teaching included the flipped classroom approach (typically defined as a type of learning where students are introduced to new material at home and practice working through it in class), a hybrid of face-to-face and online with some students attending in person and some online each day (Hybrid/Flexible or HyFlex), a synchronous online approach in which students attended real-time class sessions via video conference, and an asynchronous online approach in which students received all content through a course website and engaged with content whenever they chose (Fig 2B–2E). Each of those approaches was associated with dissatisfaction, as evident in negative values in Fig 2F, and the degree of dissatisfaction was similar across all four instructional approaches that students encountered in response to changes introduced by the pandemic (Fig 2F).

**Fig 3:**
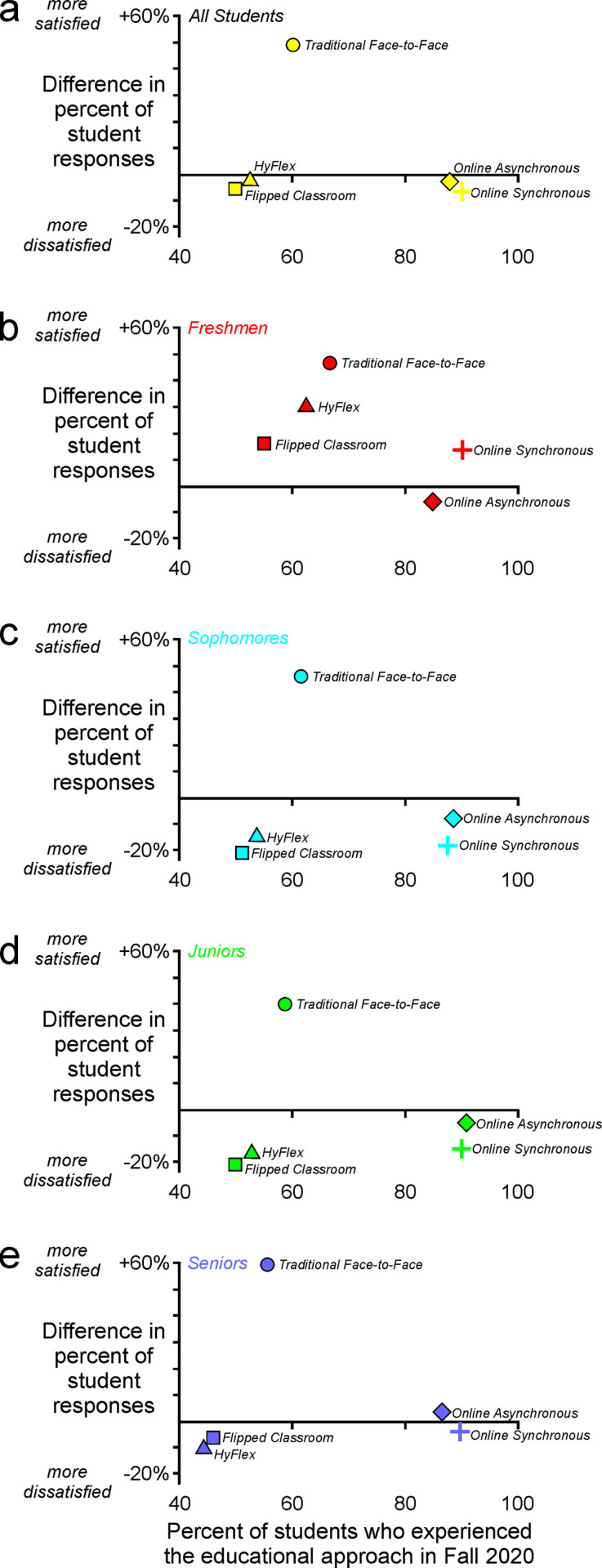
Students’ satisfaction with instructional format varied across class standings. (a) Satisfaction with instructional formats other than face-to-face was (b) greatest among freshmen students and (c-e) broadly similar among students with more advanced standing.

To further investigate the role of these instructional approaches in affecting the degree to which students reported satisfaction with their educational experience, we subdivided the responses of students according to their class standing (e.g., freshmen, sophomores) (Fig 3A–3E). Notably, freshmen (Fig 3B) tended to report greater satisfaction than students in academically older cohorts (Fig 3C–3E). The HyFlex, flipped classroom, and online synchronous approaches were each much more palatable to freshmen students than to students in any of the later years (Fig 3B–3E). As elaborated in the following sections, these differences among students with different class standing may reflect a difference in the experiences and expectations of students in their first year versus those in their later years of undergraduate education.

### Students’ satisfaction and decision to persist in their education were impacted by many factors

**Fig 4:**
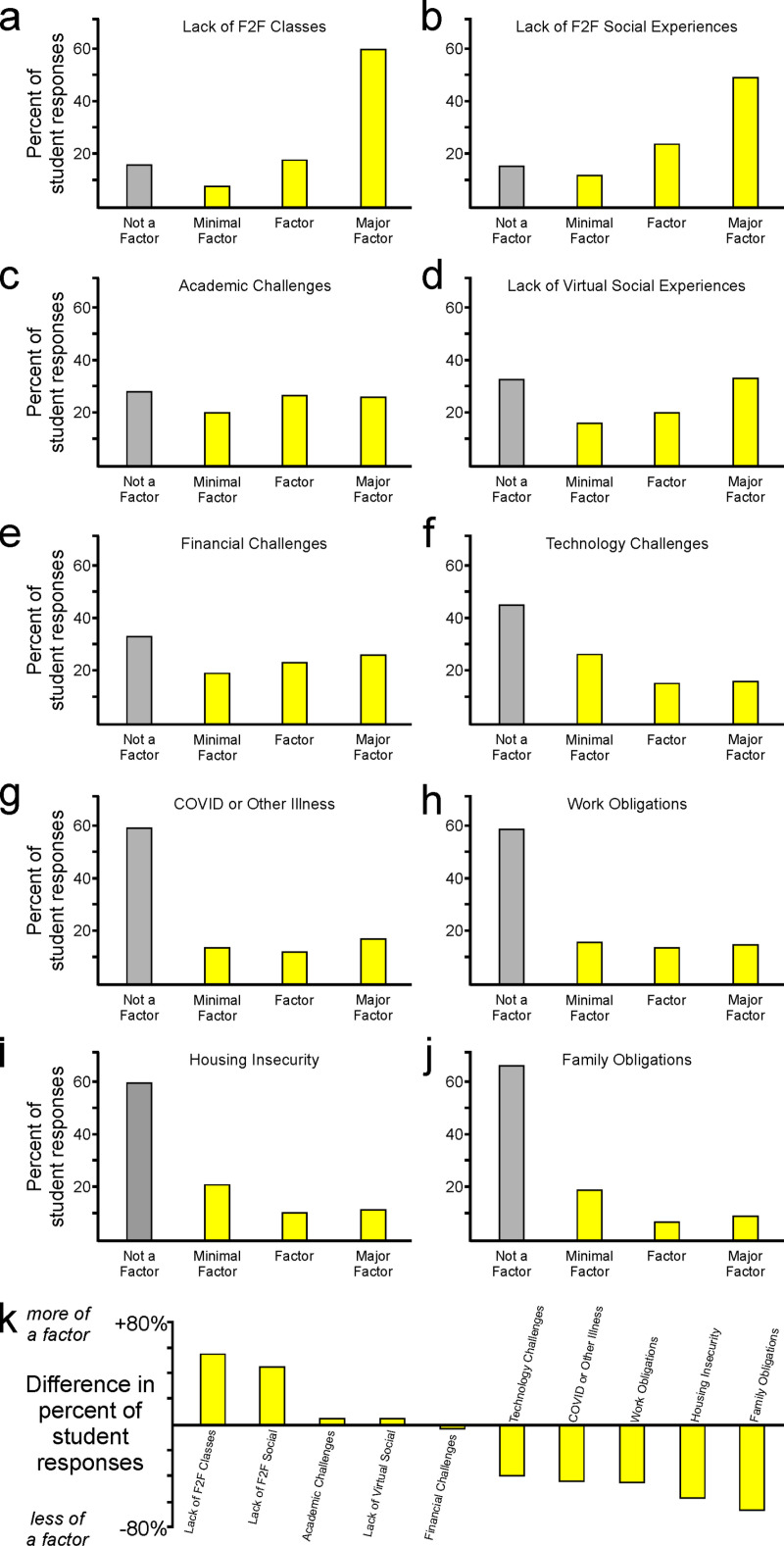
Impact of various institutional factors on students’ perception of success and well-being. (a-j) Students reported that their success and well-being was affected by a wide range of factors, with (k) lack of face-to-face educational and social experiences having the greatest impact.

Students reported that face-to-face experiences, including in-person courses and social experiences, had a major impact on their satisfaction and decision about whether to return in the following semester (Fig 4A–4B). Academic challenges, financial challenges, and virtual social experiences had some impact on their decisions, but these three factors (Fig 4C–4E) were less influential than either of the face-to-face experiences in the preceding panels (Fig 4A–4B, comparisons reported in Fig 4K). Students reported that additional factors such as technological challenges, housing challenges, personal or family illness during the pandemic, and work obligations had very little impact on their decision (Fig 4F–4J). Together, these data reveal that although many factors affected students’ decisions to persist in their education, face-to-face factors were by far the most impactful contributors to those decisions (Fig 4K).

**Fig 5:**
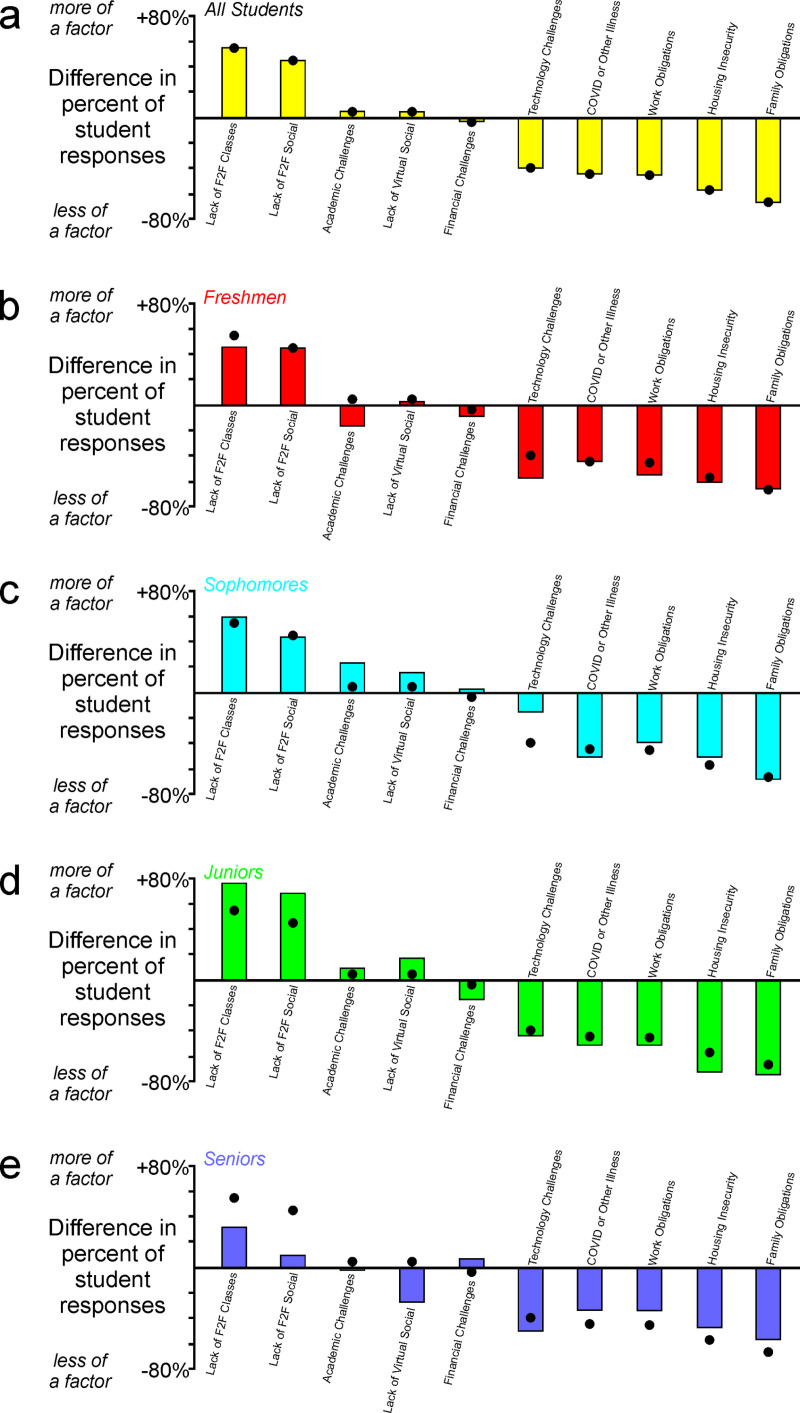
Students’ perception of success and well-being across class standings. (a-e) In contrast to students’ reports regarding satisfaction with various instructional approaches (Fig 3), there were no consistent differences in reports of impact of various factors across different academic standings.

In contrast to what we observed when we compared the degrees of student satisfaction with various educational approaches across different class standings (Fig 3), there were no consistent differences between freshmen students and other class standings in their perceived impact of various factors (Fig 5). Each class standing reported mixed results, with greater impact of some factors and a lesser impact of other factors as compared to the aggregate student response (aggregate responses are indicated by black dots in panels 5A-5E). Curiously, students with senior standing responded with less strongly held opinions than the rest of the students. Responses from students with senior standing were commonly closer to the midline than the dot indicating the aggregate response from all students (Fig 5E). This aspect of responses from senior students suggests that they felt less impacted by these factors than students in other class standings.

### Students’ perceptions of success or struggle in the pandemic experience were strongly influenced by personal and social factors

**Fig 6:**
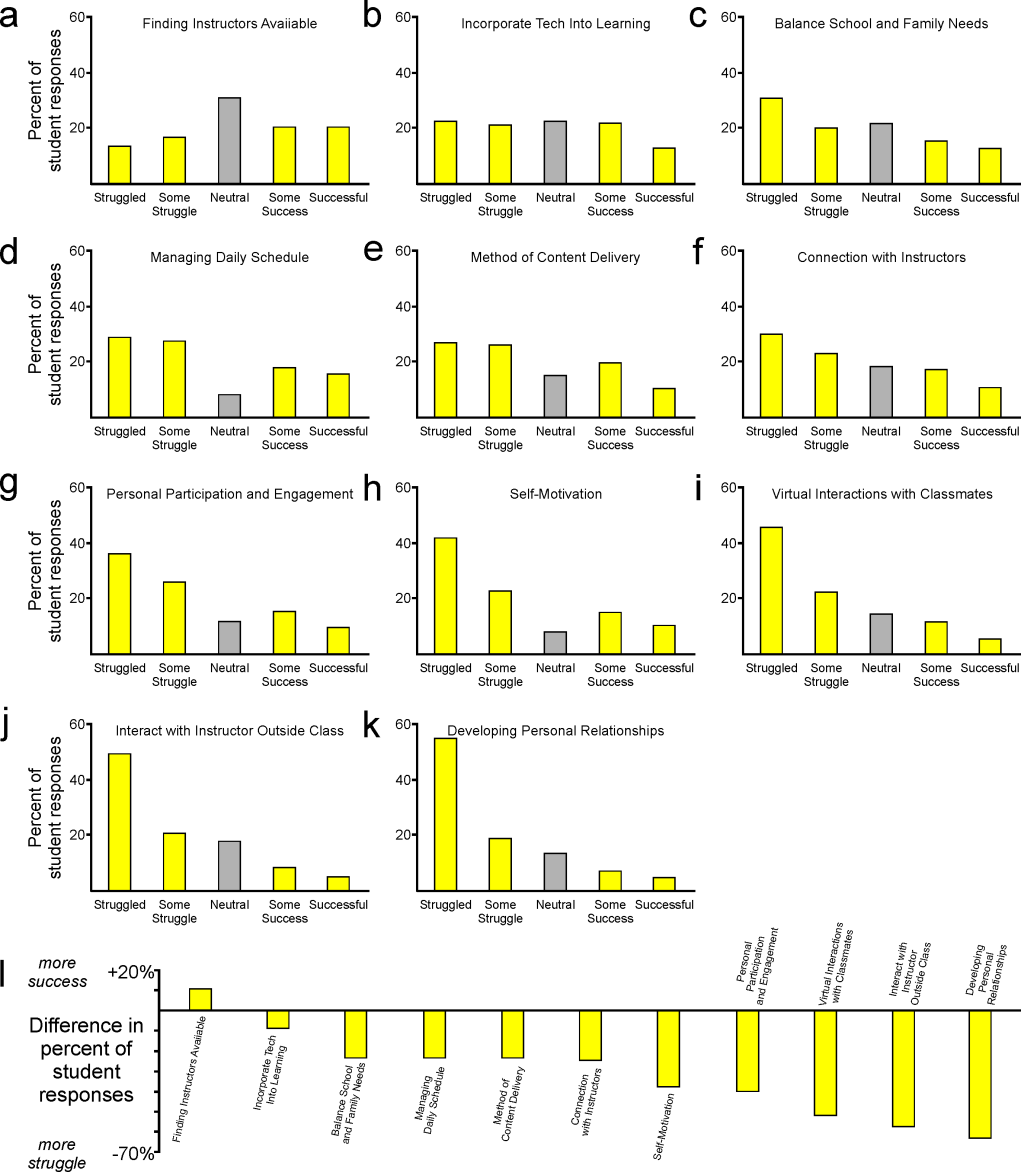
Impact of various personal factors on students’ perception of success and well-being. (a-k) Students reported that their perceived success or struggle was affected by a wide range of factors, with (k) overall success in finding instructors available but struggles in all other facets of personal and educational experience during the pandemic.

In addition to asking about students’ perceptions of educational approaches and the impact of specific factors on their decision to return in the following semester, we also asked about the role of a suite of additional factors in affecting students’ feelings of success or struggle in the academic setting. Students reported varying degrees of success in a range of factors that spanned personal approaches to motivation and time management, interactions with peers, interactions with instructors, and engagement with technological aspects of the educational experience (Fig 6A–6K). The panels in Fig 6A to 6K are arranged in a sequence that reflects students’ success or difficulty with each factor, with students reporting the greatest degree of success for the factor presented in panel 6A (finding instructors available) and the greatest degree of struggle for the factor presented in panel 6K (developing personal relationships). The data reveal only one factor (finding instructors available) for which students reported a greater degree of success than struggle, evident as a positive difference between those measures (positive value in Fig 6L). These data reveal that students experienced many challenges, but that was not due to difficulty finding their instructors available.

There were some factors for which students reported moderate degrees of struggle, and others where students reported great difficulty (Fig 6L). The data revealed three factors that were especially impactful on students’ perception of success. Those factors included aspects of interaction with peers (virtual interactions with classmates, developing personal relationships) and interaction with instructors (interaction with instructor outside of class time). These data point to the importance of interpersonal interactions as a primary driver in students’ perception of well-being and success during the pandemic and the associated transition to online learning.

Students reported very little difficulty incorporating technology into their learning experience (Fig 6L). However, students reported a greater degree of struggle in developing a connection with their instructors (Fig 6L). Students also reported great difficulty in interacting with instructors outside of class (Fig 6L). Together, these data reveal that students had little difficulty in finding opportunities to communicate with their instructors but were nonetheless challenged to develop a meaningful connection. These interactions, such as those that occur immediately after class, can be valuable contexts in which students can ask questions that they may be hesitant to ask in class, and this can allow students to receive the individualized feedback that can be an important component of their success in a course. These types of conversations can also be places where students learn about professional opportunities in their field of interest, such as scholarships or internship opportunities. Students’ frustration in their inability to forge those relationships with leaders in their degree program suggest that they were aware of those limitations, and these results further highlight the degree to which students desired face-to-face interactions.

Students reported considerable struggles in their personal participation and engagement, self-motivation, virtual interactions with classmates, and difficulty in developing personal relationships (Fig 6L). This was also frequently reflected in students’ responses to the question “what would you tell a friend about your experiences?” Many students said they would tell their friends not to come to college, or to take the semester off. Those data lend further support to the realization that students encountered many personal and social hardships in the pandemic environment. As noted earlier, students generally returned and continued in their studies in the next semester. Therefore, they may have been aware of the possible benefits of pausing their education by taking a semester off, but their perseverance suggests that they also appreciated the value of adapting to changing needs in order to continue toward timely degree completion. These realizations further emphasize the value of providing students face-to-face educational and social opportunities. These findings also highlight important aspects of the overall educational experience that institutions and instructors should seek to emphasize in future online and distance learning initiatives [24].

**Fig 7:**
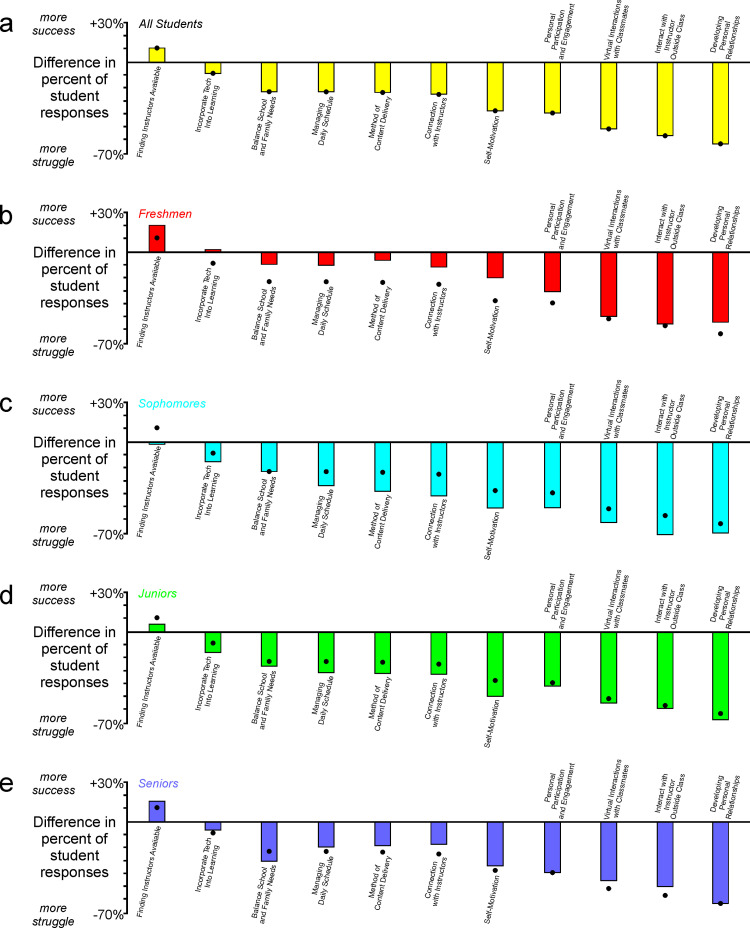
Students’ perception of importance of personal factors on success and well-being across class standings. Consistent with freshmen students’ self-reports of greater satisfaction with their COVID educational experience than students with more advanced standings (Fig 3), (a-b) freshmen students also reported greater perception of personal and educational success. (c-f) That trend was not evident in responses from students with more advanced academic standing.

When we separated the data regarding the impact of specific factors on success or struggle (Fig 6) into separate groups based on class standing, it again became apparent that freshmen tended to fare better than students with sophomore or junior standing (Fig 7A–7D). That difference is evident in responses for freshmen that are uniformly higher than the overall student responses in all cases (Fig 7B, black dots indicate aggregate responses as in Fig 5). These data indicate that freshmen typically perceived a greater degree of success in the pandemic environment (Fig 7A–7B). In contrast, responses for sophomores and juniors were the same as or lower than the overall student response in all cases, indicating that those groups perceived a greater degree of struggle (Fig 7C–7D). Responses from students with senior standing were mixed but were generally higher than the overall student response (Fig 7E). As considered in detail in later sections, these and other data suggest that freshmen and senior students may have been impacted by the pandemic in different ways and to different degrees than students with sophomore or junior standing.

**Fig 8:**
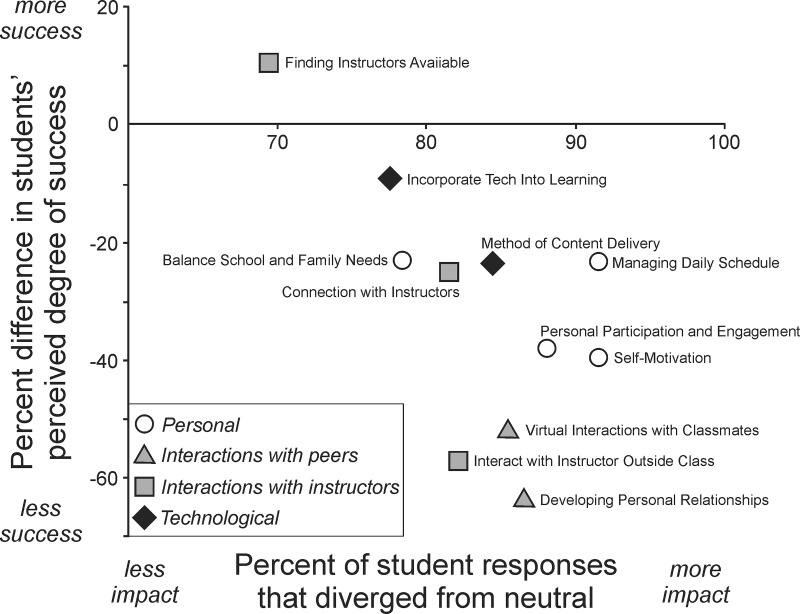
Social and personal factors affecting students’ perception of success and well-being. Students felt most successful regarding the factor that they also felt was least impactful on their experience. Technological challenges were not a major obstacle to their success, but students felt most strongly impacted and least successful regarding social interactions and personal connections with peers and instructors.

The possible answers to the question described in Figs 6 and 7 included indicators of perceived success or perceived struggle, and they also included an option of “neutral”. A neutral response can be interpreted as an indicator of an opinion that is not strongly held, whereas the other responses can be interpreted as indicators of more strongly held positive or negative opinions. From quantification of the number of neutral responses for each factor, we can identify factors that students perceived as relatively less impactful on their experience (many neutral responses) versus factors that students perceived as being more strongly impactful on their experience (few neutral responses). We used that metric to plot data along the x-axis of Fig 8, and we compared those data against students’ perceptions of how successful they were in those same factors (data along the y-axis are the same as the overall population data plotted in Fig 6L). Students felt most successful regarding the factor that they also felt was least impactful on their experience (finding instructors available, top left point in Fig 8). Interestingly, students reported that technological challenges were not a major obstacle to their success in the socially distanced and primarily online environment (filled diamonds in Fig 8). The most impactful factors (three farthest right points in Fig 8) related to aspects of the students’ individual contexts and choices. These data reveal that students were aware of and were negatively impacted by their personal struggles, but additional analyses indicate that they felt even less successful in facets of their experience that involved social interactions.

The points that are potentially of greatest concern are those that reside at the bottom of the distribution in Fig 8. Those points represent the factors where students held strong opinions that those factors were very impactful in hindering their success. These factors included difficulty developing personal relationships, difficulty interacting with classmates, and difficulty interacting with instructors outside of class. These data provide a striking summary of the value that students place on meaningful in-person interactions with their peers and with their instructors, as students clearly perceive those interactions to be vital components of their well-being and success in the undergraduate educational environment.

## Discussion

### Students remained motivated and resilient even in the midst of significant challenges

These survey data were collected during the Fall 2020 semester in some of the most challenging times during the COVID-19 pandemic. Adaptations to the educational experience that were made suddenly in response to emerging challenges in the Spring 2020 semester had by that time become more enduring aspects of students’ academic and personal lives [18]. These data are necessarily impacted by the fact that participants were answering months after the onset and immediate impact of changes associated with the pandemic, but the study benefitted from the fact that the survey was distributed after students had time to adapt and reflect on their experiences. Other possible sources of concern such as sample bias were minimized by surveying the entire undergraduate community, but insights were likely biased nonetheless by response bias. The results reported here reflect the contents of a survey developed and reviewed to ensure that defined objectives were addressed, but questions were not subjected to external review. Our survey was distributed months after the end of the semester when the pandemic began to ensure that student’s experiences and impressions were fresh in their memory.

Students expressed significant degrees of personal dissatisfaction and struggle [8–10,16–18,26–29], yet they remained largely resilient and persevered in pursuit of their degrees. The data also reveal that students typically didn’t blame the institution for those unusual challenges. Students apparently did not think that the situation was somehow better at other institutions, as students did not tend to transfer to other schools. Instead, students clearly appreciated that these anomalous events were part of a much larger adaptation of educational approaches worldwide, and students were generally willing to be patient and work cooperatively with efforts enacted by their institutional partners in their educational journey.

Students’ persistence even in the face of many perceived challenges was also evident in their return to school in the following semester. Many students were largely undeterred in their progress toward degree completion, as evident in their stated intention to return and then their actual return in Spring 2021. Even among those that were most unsettled by the impact of the pandemic (responses of undecided or no intention to return), retention was higher than would have been expected from their initial responses in the survey. Together, these data reveal that students’ motivation to succeed was quite resilient in the face of the pandemic challenges.

### Students perceive face-to-face interactions as a valuable component of their educational experience

Students reported the greatest degree of satisfaction with courses offered in the traditional face-to-face approach. This preference could have emerged for one or more reasons. First, that approach is the way that most courses at our institution were offered prior to the changes associated with the pandemic. Students might simply prefer the format that is most familiar to them and where they have developed effective ways of engaging with content, engaging with instructors and peers, and managing their time effectively. Preference for the familiar could also explain students’ dissatisfaction with other formats such as flipped classroom, HyFlex, and synchronous or asynchronous online education, but several factors argue against simply a departure from familiarity as the primary generator of student dissatisfaction. Even before the onset of the pandemic, an increasing number of courses at our institution were incorporating online content into a partially flipped format, and addition of online degree programs is also making online synchronous education more common. If dissatisfaction arose from simply engaging with novel approaches, one might expect that flipped and synchronous online formats would be more broadly preferred than the less familiar online asynchronous format. Such a trend was not evident in the data. Together, these observations led us to consider additional reasons why students might express greater preference for face-to-face approaches.

The COVID-19 pandemic led to the enforcement of social distancing requirements [30–32]. In our classrooms, this meant that each student was required to be separated by a minimum safe distance of six feet. This necessarily limited the number of students that could be in a room together, and that had the secondary consequence that only sections with small class sizes could be present in a room together. This leads to another possible factor contributing to students’ preference for face-to-face instruction. Small class sizes are more typical of advanced and specialized undergraduate courses, and large class sizes are typical of broadly relevant introductory courses. Given these differences in enrollment and the limited number of rooms on our campus that can accommodate even moderately large sections when social distancing requirements are in place, advanced undergraduate courses were among the most likely to meet together after those requirements went into effect. With the data we collected in this survey, students’ satisfaction with courses that persisted in the face-to-face paradigm could not be distinguished from their satisfaction with advanced courses that had small enrollment numbers. Those advanced courses were also likely quite relevant to their personal interests and degree progress. Data from our institution and many others point to small class sizes as a predictor of students’ satisfaction and success in a course, suggesting that small class sizes may also be an important factor at work in these results. Future extensions of this investigation will control for class sizes in order to identify the degree to which specific educational approaches are more or less beneficial for the well-being of our students.

### Students with different class standings reported different perceptions of pandemic impact

One might expect that if simple departure from familiarity was the primary driver of dissatisfaction, then students with different academic standings would be impacted in similar ways since each group had many years of in-person K-12 education prior to the changes that accompanied the pandemic. That possibility was not supported by the evidence. In fact, students with different class standings reported different reactions to pandemic-related changes, with freshmen reporting greater satisfaction than students in academically older cohorts. This suggests that a source of dissatisfaction may have been a change relative to prior undergraduate experience rather than a change relative to a lifetime of grade-school experience. A negative reaction to involuntary change would be quite understandable, as strategies that students developed in their prior undergraduate classes had to be altered, and this may have caused acute frustration and difficulty [8–10,17]. By virtue of their relative newness in the collegiate environment when the survey was administered (Fall semester), freshmen had little or no basis for comparing the contexts they encountered during the pandemic versus any previous undergraduate experience. The zeal that commonly accompanies the beginning of undergraduate life may have provided freshmen with flexibility and resilience that at least partially buffered them from frustrations that older students experienced. Incoming students may also have benefitted from support through an in-person First Year Seminar course during the first half of the Fall semester in which they received help in adjusting to college and new course formats. These targeted support opportunities may have alleviated some of the stress and frustration in a challenging freshman year.

When we compared freshmen to other classes with regard to their satisfaction with different instructional approaches and their perceptions of success or struggle in the pandemic setting, differences emerged between freshmen and students in academically older cohorts. In contrast, when we compared freshmen to other classes regarding the degree to which specific factors were impactful in their decision to persist in their undergraduate education, no clear differences emerged. When combined with the follow-up observation that students with freshmen standing were retained in proportions that were indistinguishable from those of students with sophomore or junior standing, these data suggest that students based their decision to return more on their perceived success rather than their perception of personal satisfaction or the efficacy of any specific instructional format.

In further consideration of the possible impact of specific factors on the success of students with different class standing, our analyses revealed that students with senior class standing responded with less strongly held opinions than students from other classes. This suggests that students with senior standing did not feel that those factors were as impactful as did students that were less advanced in their academic standing. Senior students may have been more strongly motivated by the prospect of degree completion and their relatively short path to that goal than by any of the factors that were addressed in the possible answers for that portion of the survey.

### Students were impacted by personal and social factors much more than external logistical factors

As evident throughout the results of this study, students reported that face-to-face experiences had a major impact on their satisfaction and decision of whether to return [see also 19,33]. Students reported that factors outside of that personal and social sphere, such as technological challenges, financial challenges, housing insecurity, personal or family illness, work obligations, and the need to balance the demands of personal and academic life, were much less impactful in shaping their experiences and perceptions. This relegation of such important factors into a less influential status suggests that students either avoided those challenges or fared well in adapting their behavior to accommodate them.

It is especially noteworthy that students reported technological challenges as not having a major impact [34–36], while personal and social factors were much more impactful [8–10,19,35,37,38]. Students very likely encountered varying degrees of technological difficulties, ranging from mild interruptions of a video or audio stream during class to complete inaccessibility of online content because of more severe interruptions of service [35,36]. Nonetheless, students reported that technological challenges were not a major factor in their decision of whether to return, and they were not associated with strong feelings of struggling in the academic setting. This further emphasizes the degree to which face-to-face factors are important, as their absence was felt much more acutely and unpleasantly than the irritations that students may have experienced due to difficulties engaging with content in a digital context.

### Building on these insights in future efforts to enhance student well-being and success

Our data point to specific ways in which the insights gained in this survey should be investigated more thoroughly to further enhance online educational efforts. Specifically, future extensions of this work should investigate not only the degree to which students report personal satisfaction and feelings of success or struggle, but also the degree to which those perceptions are related to learning outcomes such as the final grades achieved by those same students during the semester in which they completed the survey [39–42].

Future efforts to extend this work should also investigate the degree to which pandemic-related changes may have impacted different groups in different ways. At all times, and especially during circumstances that may impair student success and progress toward timely graduation, colleges and universities must thoughtfully assess the obstacles that students may face. This is especially true for underserved or underrepresented groups, including first-generation, minority, second language learners, and economically disadvantaged students. Institutions should use that information to develop targeted strategies to help improve the well-being and success of students who belong to those groups [15,43,44].

In further support of the value of future efforts to investigate impacts on specific groups, our data suggest an opportunity to help first-generation students at our institution. Approximately 30% of our respondents identified themselves as first-generation. Institutional data reveal that first-generation students are more reliant on financial aid and display lower rates of retention and persistence to graduation as compared to continuing-generation students. Specifically, first-generation students at our institution have an 11% lower average persistence rate to their second year than their continuing-generation peers, and 43% of first-year students who receive academic probation are first-generation. Consistent with those observations, preliminary analyses reveal that first-generation respondents experienced greater financial challenges, greater complications due to work obligations, and greater challenge balancing family and academic responsibilities. Institutional data reveal that the number of first-generation students that received a failing grade or a grade that did not enable them to use that class as a prerequisite for continued degree progress increased by four percent, while continuing generation students experienced little impact on that same metric. First-generation students also reported greater impact of COVID illness on their health and that of their family members. Such obstacles are common for first-generation students and underserved groups across many institutions [45–47]. These data highlight the degree to which efforts to support specific groups through targeted initiatives can make a powerful difference in students’ ability to persist and thrive in higher education [15,48–52]. A detailed analysis of the impact of the pandemic and associated changes on each of many specific groups (e.g., first-generation vs. continuing generation, comparisons by sex and gender, enrolled as freshmen vs. transfer students, full-time vs. part-time, engaging with classes online vs. in person, etc.) is beyond the scope of this initial investigation, and it must be an important future goal for our institution and others as we assess and eliminate obstacles to student success.

## Conclusions

The data in this study reveal that students encountered personal and social hardships in the pandemic environment. These insights reveal the value of providing students face-to-face educational and social opportunities early and often, and they point to specific aspects of the educational experience that should be a primary consideration as institutional leaders seek to develop new online and distance learning initiatives. Specifically, students indicated that interpersonal relationships, between themselves and their peers as well as between themselves and instructors, are primary components of their success and satisfaction in the undergraduate educational environment [18,43,44,53–56]. These personal interactions are not only important for social connection but also for supporting student achievement [57]. In-person interactions may not be possible if students in an online program are broadly dispersed, but occasional local or online meetups may be mechanisms through which institutions can facilitate the forming and deepening of these valuable relationships [53,58]. Consistent with our observations, other studies have indicated that not all students reported a negative impact of the lockdown. In fact, approximately one quarter of adolescents surveyed in the United Kingdom reported that their lives were better in the lockdown [59]. Together, these results further emphasize the degree to which specific groups may benefit from different types of approaches in online education.

In response to insights that emerged from this survey of student well-being and success, our institution has implemented a program to help all arriving students (freshmen or transfer students) forge new relationships and acclimate themselves to the collegiate academic environment. This program spans five days in the week before Fall classes begin (“Saddle Up” program implemented for the first time in Fall 2022). Students live together in the dormitories, attend orientation sessions to ensure their awareness of a wide range of on-campus resources, receive coaching in time management and academic success, and attend daily sessions of a simulated college course. To incentivize participation and recognize the value of these learning experiences, students who complete this onboarding experience receive one credit hour toward graduation. Students are evaluated only based on participation, with no academic evaluation of their performance in the simulated course. Students also receive mentoring from instructors and older student peer mentors regarding strategies that are generally beneficial or that have worked for them in their development. Together, these experiences are meant to provide a low-stakes environment where students can explore the efficacy of different strategies without fear of consequence if they encounter an unsuccessful approach along the way. This time also introduces students to faculty members who can remain mentors for them throughout their time at the institution and beyond. Equally important in light of the value that students place on personal relationships, this program also provides an opportunity for students to form new friendships among members of their residence hall, their simulated course, and throughout their cohort of new students arriving on campus. This and other approaches on our campus will continue to be assessed and revised as we seek to develop holistic approaches to benefiting students’ well-being and academic success.

## Supporting information

S1 File Survey that was distributed to students as the instrument used to collect the data described in this study. (DOCX)
