# Supplementary material for: Student perceptions of COVID-19 challenges affecting student motivation, well-being, and success in undergraduate education
Source: PLoS One. 2025 Jun 2;20(6):e0324832. doi: 10.1371/journal.pone.0324832 (PMC12129160; doi:10.1371/journal.pone.0324832)
Supplement: S1 File — (DOCX) [file pone.0324832.s001.docx]

Feedback on UW Fall Semester

Start of Block: Introduction

Q1.1 Thank you for taking the time to complete this survey.  All of your responses will be kept anonymous and strictly confidential.  At the end of the survey, there is a voluntary question in which we ask for your name and contact information.  That information will not be included in our analysis, and we will use it only for purposes of contacting students who may express a need or a desire for follow-up on the issues addressed in these questions.


Do you consent to allowing the University of Wyoming and members of the Student Success Coalition to collect and analyze your anonymous and confidential responses?

- Yes (1)
- No (2)

Skip To: End of Survey If Q1.1 = 2

End of Block: Introduction

Start of Block: UW Plans

Q2.1 Will you return to UW in the spring?

- Yes (1)
- No (2)
- Undecided (3)
- No, I graduate this semester (4)

Display This Question:

If Q2.1 = 2

Or Q2.1 = 3

Q2.2 Consider the following possible reasons for not returning in the spring, or being undecided about not returning, and select on the scale how much or little that is a factor in your decision.

|  | Not a factor for not returning (1) | A minimal factor for not returning (2) | A factor for not returning (3) | A major factor for not returning (4) |
| --- | --- | --- | --- | --- |
| academic challenges (1) |  |  |  |  |
| technology challenges (2) |  |  |  |  |
| lack of face-to-face classes (4) |  |  |  |  |
| availability and/or quality of ***face-to-face*** social/extracurricular experiences (8) |  |  |  |  |
| availability and/or quality of ***virtual*** social/extracurricular experiences (9) |  |  |  |  |
| financial challenges (3) |  |  |  |  |
| housing insecurity (7) |  |  |  |  |
| work obligations (5) |  |  |  |  |
| COVID-19 or other illnesses (6) |  |  |  |  |
| family obligations (11) |  |  |  |  |
| other: please specify (10) |  |  |  |  |

Q2.3 Do you plan to eventually graduate from UW?

- Yes (1)
- No, hope to transfer (2)
- No, don't anticipate graduating (3)
- Unsure (4)

End of Block: UW Plans

Start of Block: Successes and Challenges

Q3.1 Below are several aspects of this fall's academic experience.  Consider your own experience this semester, and indicate which point on the 5 point scale most closely reflects how much you struggled or were successful with each of these aspects.

|  | Struggled (1) | Somewhat struggled (2) | Neutral (3) | Somewhat successful (4) | Successful (5) |
| --- | --- | --- | --- | --- | --- |
| Managing a daily schedule that included online classes. (1) |  |  |  |  |  |
| My personal engagement and participation during online classes. (2) |  |  |  |  |  |
| Finding Professors' available for academic help (e.g. before/after class, office hours). (4) |  |  |  |  |  |
| Establishing a helpful connection with my Professors. (9) |  |  |  |  |  |
| Professors' method of content delivery within the online courses (e.g., lecture vs discussion, etc) (3) |  |  |  |  |  |
| Incorporation of new online technologies into classes and learning. (8) |  |  |  |  |  |

Q3.2 Below are several factors that impact college life and academics.  Consider your personal experience this semester, and indicate which point on the 5 point scale most closely reflects how much you struggled or were successful with each of these aspects.

|  | Struggled (1) | Somewhat struggled (2) | Neutral (3) | Somewhat successful (4) | Successful (5) |
| --- | --- | --- | --- | --- | --- |
| Self motivation (1) |  |  |  |  |  |
| Virtual interactions with classmates (2) |  |  |  |  |  |
| Developing personal relationships. (3) |  |  |  |  |  |
| Opportunities to interact with Professors outside the classroom. (7) |  |  |  |  |  |
| Balancing family obligations with UW commitments (e.g., academics, extracurriculars, etc.) (8) |  |  |  |  |  |

| 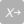 |
| --- |

Q3.3 Please indicate your level of satisfaction with the learning quality you experienced in each of the following formats. If you have never worked in one of the formats, please exclude that from your rankings.

|  | Very dissatisfied (1) | Dissatisfied (2) | Satisfied (3) | Very satisfied (4) | Did not experience (0) |
| --- | --- | --- | --- | --- | --- |
| Traditional, in person face-to-face (meeting regularly in a classroom) (1) |  |  |  |  |  |
| Flipped classroom of in person class meetings and online content completed prior to class (5) |  |  |  |  |  |
| HyFlex/Hybrid: combining online and in person (meeting in person some days of the week and online the other days) (2) |  |  |  |  |  |
| Fully online synchronous (e.g., Zoom) (4) |  |  |  |  |  |
| Fully online asynchronous (e.g., prerecorded lectures) (6) |  |  |  |  |  |

End of Block: Successes and Challenges

Start of Block: Fall Semester Feedback

| 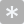 |
| --- |

Q4.1 What would you tell a friend about your experiences at UW this semester?

________________________________________________________________

________________________________________________________________

________________________________________________________________

________________________________________________________________

________________________________________________________________

| 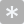 |
| --- |

Q4.2 What tips would you give a friend about being successful at UW during the COVID-19 pandemic?

________________________________________________________________

________________________________________________________________

________________________________________________________________

________________________________________________________________

________________________________________________________________

| 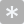 |
| --- |

Q4.3 What were the most helpful approaches that you developed to facilitate your own study habits and educational success this semester?

________________________________________________________________

________________________________________________________________

________________________________________________________________

________________________________________________________________

________________________________________________________________

| 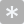 |
| --- |

Q4.4 What were the most helpful approaches that your instructors developed to help you succeed in your courses this semester?

________________________________________________________________

________________________________________________________________

________________________________________________________________

________________________________________________________________

________________________________________________________________

End of Block: Fall Semester Feedback

Start of Block: Demographics

Q5.1 Which best describes your living situation during Phase 3 (currently) of the fall semester?

- Residence Halls (1)
- UW Apartments (2)
- Fraternity/Sorority Housing (3)
- Off campus housing within walking distance of campus (4)
- Off campus housing within commuting distance of campus (5)
- Off campus--further than commuting distance, but within Wyoming (6)
- Off campus outside Wyoming (7)
- in transition/homeless (8)

Q5.2 Are you a first generation college student (no immediate family member has graduated from college)?

- Yes (1)
- No (2)

End of Block: Demographics

Start of Block: Student Information

Q6.1 Please enter your first and last name.

________________________________________________________________

| 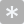 |
| --- |

Q6.2 Please enter your W# using the following format: W01234567

________________________________________________________________

| 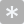 |
| --- |

Q6.3 Please enter your phone number using the following format: 307-555-5555

________________________________________________________________

| 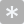 |
| --- |

Q6.4 Please enter your preferred email address.

________________________________________________________________

End of Block: Student Information
